# Nutritional Deficiencies and Phospholipid Metabolism

**DOI:** 10.3390/ijms12042408

**Published:** 2011-04-06

**Authors:** María S. Gimenez, Liliana B. Oliveros, Nidia N. Gomez

**Affiliations:** Department of Biochemistry and Biological Sciences, Faculty of Chemistry, Biochemistry and Pharmacy, National University of San Luis, IMIBIO-San Luis CONICET, Chacabuco and Pedernera, 5700-San Luis, Argentina; E-Mail: ngomez@unsl.edu.ar

**Keywords:** phospholipids, unsaturated fatty acids, vitamins, oligoelements

## Abstract

Phospholipids are important components of the cell membranes of all living species. They contribute to the physicochemical properties of the membrane and thus influence the conformation and function of membrane-bound proteins, such as receptors, ion channels, and transporters and also influence cell function by serving as precursors for prostaglandins and other signaling molecules and modulating gene expression through the transcription activation. The components of the diet are determinant for cell functionality. In this review, the effects of macro and micronutrients deficiency on the quality, quantity and metabolism of different phospholipids and their distribution in cells of different organs is presented. Alterations in the amount of both saturated and polyunsaturated fatty acids, vitamins A, E and folate, and other micronutrients, such as zinc and magnesium, are discussed. In all cases we observe alterations in the pattern of phospholipids, the more affected ones being phosphatidylcholine, phosphatidylethanolamine and sphingomyelin. The deficiency of certain nutrients, such as essential fatty acids, fat-soluble vitamins and some metals may contribute to a variety of diseases that can be irreversible even after replacement with normal amount of the nutrients. Usually, the sequelae are more important when the deficiency is present at an early age.

## 1. Introduction

The nutritional adequacy of dietary components depends on both their amount and bioavailability in the diet. Insufficient dietary intake or excessive loss of macro- and micronutrients strongly affect the phospholipids of cellula!r membranes in all living organisms, leading to pathophysiological situations of clinical importance in human health.

The fatty acid composition of membrane phospholipids is likely to be affected by the exogenous fatty acids from the diet or by altered activities of lipid-metabolizing enzymes such as fatty acid desaturases [1,2]. Also, some vitamins and trace elements that occur in the human diet are known to participate in phospholipid homeostasis in different cells. The objective of this review is to provide a current overview of the impact of some micronutrient deficiencies of public health significance in phospholipid content, distribution and metabolism in different tissues. The effects of the following micronutrients: (1) fatty acids; (2) fat-soluble vitamins A and E, with emphasis on their effect on liver, which participates in the body-wide distribution of these vitamins, and on the cardiovascular system, which is particularly affected by lipid peroxidation. Folate deficiency, the most prevalent vitamin deficiency throughout the world, is also discussed. Finally, an overview of (3) dietary zinc (Zn) and magnesium (Mg) and their correlation with phospholipid homeostasis in the lung and in the cardiovascular system, respectively, is also given.

## 2. Importance of Dietary Fatty Acids on Phospholipid Distribution and Metabolism

The degree of fatty acid unsaturation in membrane phospholipids determines the biophysical properties of the membrane, which in turn influences many critical membrane-associated functions [3,4]. Depletion of polyunsaturated fatty acids (PUFAs) from membranes might be expected to alter their physical and functional properties [5,6].

### 2.1. Phospholipids in Brain

The cerebral cortex neuronal membrane phospholipids are composed of phosphatidylcholine, phosphatidylethanolamine, phosphatidylserine, and phosphatidylinositol [6]. Phospholipids contain unsaturated fatty acids, in particular docosahexaenoic acid [DHA, 22:6 (*n* − 3)], the highest levels of which are found in the retina and in the cerebral cortex. The degree of unsaturation present in neuronal phospholipids fatty acids can mediate the activities of membrane bound enzymes [7].

The best sources of w-3 DHA in the diet are seafood, algae, and especially coldwater fish and fish oil. Alfa-linolenic acid [ALA, 18:3(*n* − 3)] appears not to be efficient as a good source for DHA, since its conversion to eicosapentaenoic acid [EPA, 20:(*n* − 3)] occurs but is limited in men and the further transformation to DHA is very low [8].

In rats, dietary deprivation of w3 fatty acids has been associated with a decrease in the electroretinogram amplitude and impairment in the ability to learn a visual discrimination [9]. *In vitro*, the growth of neurites increases the demand for phospholipids, and the biosynthesis of phosphatidylcholine is stimulated in response to nerve growth factor [10]. Infants fed with milk artificial formulas exhibit differences in the fatty acid composition of the cortex neuron phospholipids as compared with breast-fed infants. Artificial formulas cause a decrease of DHA in phosphatidylserine and phosphatidylethanolamine, which has been shown to have clinical consequences particularly in preterm infants that present significantly different electroretinographic patterns indicating delayed rod photoreceptor maturation compared with those fed with human milk or given supplementary DHA [37]. Carlson *et al*. [12] have shown that DHA supplemented preterm infants have greater red blood cell phosphatidylethanolamine DHA content and better visual acuity than standard formula fed preterm infants. DHA supplemented infants performed significantly better than controls on the Bayley mental scales. It has also been shown that term infants' visual responses at five months after birth correlate with erythrocyte DHA concentrations [13].

Long-term intake of a DHA rich diet is also essential for maintaining and improving the ability of learning and memory in elderly humans. Levels of arachidonic [AA, 20:4(*n* − 6)] and docosatetraenoic acids and also DHA decrease considerably in gray matter and hippocampus phospholipids of patients with Alzheimer’s disease [14]. Changes in phospholipids metabolite levels have been correlated with neuropathological hallmarks of Alzheimer’s disease and cognitive decline. In fact, phospholipids alter the production of Aβ40, Aβ42 peptides and consequently Aβ42:40 ratios [15].

It is currently known that the fate of DHA in the brain phospholipids is also to generate a protective eicosanoid: neuroprotectin D1 (NPD1), which has a potent anti-inflammatory and neuroprotective bioactivity. NPD1 reduces Ab42 peptide release from aging human brain cells and is severely depleted in Alzheimer’s disease brain [16]. NPD1 also exerts a citoprotective effects on retina [17]. Dietary strategies aimed at reducing Aβ levels should take into account interactions of dietary components and the metabolic outcomes [15].

Using an experimental model in rat, it has been observed that percentages of both AA and docosatetraenoic acid of phosphatidylethanolamine and the level AA of phosphatidylcholine in rats fed a sardine oil diet were lower than in rats fed a palm oil diet. However, the level of DHA of phosphatidylethanolamine in the sardine oil diet group was higher than that in the palm oil diet group, and there were no significant differences in the percentages of stearic, oleic, or palmitoleic acids of phospholipids between the experimental diet groups. Adult mice fed a sardine oil diet for a long period maintained a higher level of DHA in brain stem phospholipids, higher synaptic membrane fluidity and higher maze-learning ability than animals fed a palm oil diet [18].

Furthermore, low tissue levels of (*n* − 3) PUFA in phospholipids, particularly DHA, are implicated in postpartum depression. Brain regions of parous female rats fed a diet containing inadequate (*n* − 3) PUFA exhibited a regionally specific decrease of phospholipid DHA content that was not caused by either reproductive status or ALA-deficient diet alone, indicating that this effect was due to an interaction between the diet and the physiological status. Thus, the specific neuronal systems may be differentially affected by depletion of brain DHA in the postpartum organism [19].

### 2.2. Phospholipids in Heart

Several reports indicate that consumption of w-3 fatty acids from fish oil, specifically EPA and DHA, decreases the risk of heart failure and attenuates pathologic cardiac remodelling in response to pressure overload. However, there are contrasting results in the literature regarding the benefits of PUFA supplementation for the heart. According to a recent report on the fatty acid content of phospholipids of erythrocyte membranes taken from atrial fibrillation patients, DHA in the w-3 series was significantly higher in these patients making them more susceptible to oxidative damage than controls [20].

Dietary supplementation with EPA + DHA may impact cardiac mitochondrial function and energetics through alteration of membrane phospholipids. EPA+DHA altered fatty acid composition of total mitochondrial phospholipids and cardiolipin by reducing AA content and increasing DHA incorporation [21]. Treatment with w-3 PUFAs DHA and EPA exerts cardioprotective effects, and suppresses Ca^2+^ induced opening of the mitochondrial permeability transition pore (MPTP). These effects are associated with increased levels of DHA and EPA and lower levels of AA [22]. In heart, phosphatidylethanolamine highest levels of EPA and DHA were observed following fish oil intake.

It is known that caloric restriction results in phospholipid depletion, membrane remodelling and triacylglycerol accumulation in murine myocardium. After brief periods of fasting (4 and 12 h), substantial decreases occurred in the choline and ethanolamine glycerophospholipid pools in murine myocardium. Remarkably, the selective loss of long-chain polyunsaturated molecular species was present in the major phospholipid classes thereby altering the physical properties of myocardial membranes [23].

On the other hand, epidemiological evidence indicates a strong relationship between the intake of *trans* fatty acids, which are formed during the hydrogenation process of *cis*-unsaturated fats, and the risk of coronary heart disease [24,25]. This has been associated to increased LDL cholesterol/HDL cholesterol and total cholesterol/HDL-cholesterol ratios, and increased Lp(a) lipoprotein levels when *trans* fatty acids are substituted for saturated fatty acids [26]. Also, *trans* fat inhibits the metabolic conversion of linoleic acid [18:2(*n*−6)] to AA and to other n-6 PUFA in aorta, the target tissue of atherogenesis [27]. In an atherogenic animal model, *Apo E−/−*mice fed a low *trans* fat from corn oil showed an increase of plasma HDL-cholesterol, apo A-I concentrations and the ratio of HDL-cholesterol to total cholesterol, while there was a decreases of apo B level compared with hydrogenated *trans* fat [28]. However, further studies are required in order to justify recommendations to use low *trans* fats in food processing.

### 2.3. Phospholipids in Bone

Phospholipids play an important role in the bone marrow as fatty acid reservoirs. It has been shown that fatty acids may influence bone metabolism by altering the biosynthesis of prostaglandins. In particular, (*n* − 3) PUFA increases bone formation by decreasing PGE2 biosynthesis [29]. Changes of the membrane phospholipid fatty acid composition may affect bone cell signalling and, potentially, bone mineralization. Using the fat-1 mouse, a transgenic model that synthesizes (*n* − 3) from (*n* − 6) PUFA, it was determined that mice fed with a diet containing 10% safflower oil, phosphatidylcholine phosphatidylethanolamine and phosphatidylserine fractions in the vertebrae had a significantly lower (*n* − 6)/(*n* − 3) ratio than wild-type mice. In fat-1 femurs, these fractions, along with phosphatidylinositol, also had a lower (*n* − 6)/(*n* − 3) ratio than wild-type mice. DHA was positively correlated with bone mineral density in all fractions in the vertebrae, and in phosphatidylinositol and phosphatidylserine in the femur [30].

An analysis of fatty acid composition of aspirated bone marrow in patients with hematologic diseases has revealed that oleic and palmitic acids concentrations are higher in bone marrow than in serum phospholipids. Also, oleic acid but not palmitic acid, increases the rate of alkaline phosphatase positive ST2 cells induced by bone morphogenetic protein-2 [31]. It has been proposed that all these differences may affect osteoblast differentiation in the bone marrow microenvironment [31]. However, the effects of phospholipid fatty acids on osteoblast differentiation are not yet fully understood.

### 2.4. Phospholipids in Intestine

Malnutrition induced by dietary restriction in nursing piglets severely affects the intestinal histological structure. The amount of DNA and protein, the content of cholesterol, phospholipids and triglycerides, and the relative percentages of (*n* − 6) and (*n* − 3) long chain-PUFA are lower and those of (*n* − 9) fatty acids are higher in the jejunal and ileal mucosa of protein-malnourished animals. These differences, together with those in the distribution of phospholipid species, have been associated with an alteration of the activity of membrane-bound hydrolytic enzymes [32]. Long-chain (*n* − 6) and (*n* − 3) PUFA in total mucosa, microsomes and mucosa phospholipids of jejunum of protein-energy malnourished piglets were recovered by consuming a long chain-PUFA formula. Dietary (*n* − 3) and (*n* − 6) long chain-PUFA was efficiently taken up and acylated into membrane phospholipids of jejunal cells. These fatty acids in the diet may influence the recovery of intestinal injury caused by malnutrition [32]. On the other hand, phospholipids are the main component of the lipoprotein surface, and hence a reduction in the content of intestinal phospholipids could lead to alterations of lipoprotein conformational structure and secretion [33].

Human studies indicate that fatty acid composition of the intestinal mucosa of children with active celiac disease show significant differences compared with controls. In celiac children, the level of linoleic acid is decreased, whereas that of its derivatives was elevated, indicating increased transformation of (*n* − 6) fatty acid. Furthermore, the level of Mead acid [(20:3(*n* − 9)] is increased, with an increased ratio of Mead acid to AA level, suggesting essential fatty acid deficiency. The fatty acid abnormality of intestinal mucosa in these patients was not reflected in serum values [34].

### 2.5. Phospholipids in Liver

High fat diet induces pronounced ceramide and sphingomyelin accumulation in both liver and hepatic nuclei and a greater fatty acids saturation status in these sphingolipids [35]. Furthermore, it has been observed that liver-specific loss of long chain acyl-CoA synthetase-1 alters phospholipid fatty acid composition. Small but consistent increases were determined in the percentage of 16:0 in phosphatidylcholine and phosphatidylethanolamine and of 18:1 in phosphatidylethanolamine and lisophosphatidylcholine, whereas concomitant decrease were seen in 18:0 in phosphatidylcholine, phosphatidylethanolamine, phosphatidylserine and lisophosphatidylcholine [36].

It has been shown that low (*n* − 6)/(*n* − 3) fatty acid ratio, with fish- or flaxseed oil in a high fat diet, improves plasma lipids and beneficially alters the liver fatty acid composition of phospholipids in mice. The levels of EPA and DHA in liver phospholipids were significantly increased in both fish and flax groups as compared to the control group, with more marked increases in the fish group [37].

Diet (*n* − 3) PUFA content, and parity, affect liver and erythrocyte phospholipid fatty acid composition in rats. The effects of sequential pregnancies on the phospholipid fatty acid compositions of the maternal liver and erythrocytes have been determined in female rats fed diets containing ALA, ALA and preformed DHA (ALA + DHA), or minimal ALA (low ALA). Virgin females, fed the diets for commensurate durations, served as a control for reproduction. A significant interaction between diet, reproductive status, and duration of treatment was detected only for DHA. Primiparous dams fed the ALA and low ALA diet showed a decreased liver DHA content compared with virgin females fed the ALA diet. Liver DHA did not decrease further after additional reproductive cycles. Liver DHA content was unchanged in porous dams fed the ALA + DHA diet, but virgin females fed this diet exhibited a 50% increase in liver DHA after 13 weeks of treatment. Similar changes were observed in erythrocytes [38]. Liver phospholipid concentration was 1.27-fold lower with fish protein than with casein, respectively. The fish protein diet lowered the AA proportion and the ratio of AA to linoleic acid in liver microsomal phospholipids, which was due to the reduced microsomal delta6(w-6) desaturation activity [39].

Using the paradigm of Weindruch *et al*. [40] different levels of calorie restriction (CR) (125, 85, 50, or 40 kcal/week for 1, 3, and 6 months) have been examined in mice. Changes in the fatty acid composition of phospholipids from liver, kidneys, heart, brain, and skeletal muscle were observed following 1 month of calorie restriction [41].

### 2.6. Phospholipids in Serum and Plasma

Several fatty acids are suitable biomarkers for usual dietary intakes. Serum oleic acid has been directly associated with olive oil, linoleic acid with sunflower oil, pentadecanoic acid with dairy products, (*n* − 3) long chain-PUFA with fatty fish, and *trans*-monounsaturated fatty acids with manufactured foods [42,43].

Different pathologies, such as pancreatitis, cancer, diabetes, coronary heart disease, among others, can be associated to changes in plasma or serum fatty acids phospholipids. It has been communicated that patients with alcoholic pancreatitis showed an increase of monounsaturated fatty acids and a decrease of AA in plasma phosholipids compared to controls, while no differences were observed in values of EPA and DHA or in the ratio of (*n* − 3) to (*n* − 6) long chain-PUFA [44].

Positive associations of serum phospholipids with ALA and negative associations with the ratio of linoleic acid to ALA have been related with the risk of prostate and breast cancer [45,46].

Furthermore, positive associations with diabetes were observed for stearic acid and total saturated fatty acids in plasma phospholipid whereas an inverse association was reported for linoleic acid [63]. In addition, hydrogenated *trans* fatty acid intake has been positively associated with waist circumference and glycated hemoglobin [48].

The apparently strong findings for an association between plasma or serum phospholipids fatty acids and different pathologies should be confirmed by nutritional-epidemiologic studies in different populations.

## 3. Vitamin A: Its Effects on Phospholipid Metabolism

Vitamin A is an essential fat-soluble nutrient for vertebrates. Dietary fat intake is extremely low in most communities with vitamin A deficiency [49]. Since animals lack the capacity for *de novo* synthesis of retinoids, they have an obligatory need to obtain retinoid from the diet as either preformed vitamin A (all-*trans*-retinol) or provitamin A carotenoids. In particular, experimental evidence indicates that phospholipid metabolism is altered in different tissues of the vitamin A-deficient rats, which suggests a role for vitamin A in maintaining the integrity of the cell membranes.

Recently, a beneficial effect on the vitamin A status of rural Bangladesh women and their children has been shown when supplementing women with fat (20 ml soybean-oil/day) from mid-late pregnancy until six months postpartum [49].

### 3.1. Vitamin A Deficiency on Liver Phospholipid Metabolism

It is well established that liver phospholipid content decreases in rats with vitamin A deficiency [50]. In contrast, it increases in guinea pig with the administration of 100,000 U.I. of vitamin A for seven days [51]. The lower content of total phospholipid in liver of vitamin A-deficient rats has been associated to a lower synthesis of phosphatidylcholine and a lower availability of fatty acids [52]. The incorporation of [^14^C]-acetate into saponifiable lipids and the activity of acetyl-CoA carboxylase decreases in the hepatic tissue [52]. The low fatty acid synthesis and the depletion of retinoids and retinoid X receptors (RXR) found in the vitamin A-deficient liver could explain the low mRNA expression of the hepatic transcription factor peroxisome proliferator-activated receptor alpha (PPARalpha) which has a pivotal role in the transcriptional regulation of genes involved in cellular lipid metabolism [52].

Additionally, a significant decrease in total phospholipid content occurs in liver mitochondria of vitamin A deficient-rats giving an increased cholesterol/phospholipid relation, which suggests that membrane fluidity can be altered. This, together with the fact that mitochondrial cardiolipin content decreases, could lead to mitochondrial dysfunction. Cardiolipin is recognized to be an essential phospholipid in eukaryotic energy metabolism and in maintaining mitochondrial structure and function. A direct relationship between cardiolipin loss and cytochrome c released from the mitochondria has been identified as an initial step in the pathway to apoptosis [53]. In addition, deprivation of vitamin A for three months has been shown to induce lipoperoxidation in rat liver [54]. Dysfunctional hepatocytes, with a loss in mitochondrial cardiolipin and a decreased mitochondrial membrane potential is associated with mitochondrial oxidative stress and pathogenesis and progression of different liver diseases [59]. The mitochondrial inner membrane enzyme complexes of the electron transport chain also bind phosphatidylcholine and phosphatidylethanolamine, which are required for the optimum activity of complexes I and III [60].

### 3.2. Vitamin A Deficiency on Heart Phospholipid Metabolism

Lipid metabolism is significantly modified in the heart of vitamin A-deficient rats. In the left ventricle the content of total phospholipids increases due to an increase of phosphatidylcholine, phosphatidylethanolamine and phosphatidic acid, while cardiolipin, sphingomyelin, and lisophosphatidylcholine decrease compared to the control-fed group. Vitamin A refeeding partly restores sphingomyelin and lisophosphatidylcholine contents, and completely restores cardiolipin, phosphatidylcholine, phosphatidylethanolamine and phosphatidic acid proportions to control values [57]. Changes in the content of phosphatidylcholine and sphingomyelin in the heart ventricle are explained by alterations in their endogenous synthesis. In contrast, it has been shown that the administration of 33 mg of vitamin A for two days results in a decrease of total phospholipids and phosphatidylcholine contents in hearts of rats, due to the impaired synthesis of phospholipids by the diphosphocholine-ethanolamine pathway [58]. It is necessary to consider that the maintenance of a constant sphingomyelin to cholesterol ratio in membranes is important to support the critical function carried out by rafts and related membrane structures [59]. Changes in the contents of lisophosphatidylcholine, which is known to stimulate the efflux of ^45^Ca^2+^ from freshly adult rat cardiomyocytes [60], and cardiolipin, which is essential for energy metabolism and in maintaining mitochondrial structure in the heart [61], may affect the mitochondrial function.

Furthermore, vitamin A deficiency induces a decrease of linoleic acid and a high level of saturated long-chain fatty acids, 16:0 and 18:0, in the heart mitochondrial phospholipids [57]. Since linoleic acid is the main fatty acid of cardiolipins [62], and palmitate has been shown to decrease the content of mitochondrial cardiolipin in rat neonatal cardiomyocytes [63], the significant decrease of cardiolipin content and the increase of its precursor, phosphatidic acid, in the heart tissue of vitamin A-deficient rats [57] could indicate an inhibition of cardiolipin synthesis. In addition, mitochondrial dysfunction by cardiolipin oxidative damage has been mainly ascribed to a specific loss in mitochondrial content of cardiolipin. It is known that reactive oxygen species (ROS) affect the activity of heart mitochondrial complex III leading to mitochondrial dysfunction via cardiolipin oxidative damage [56]. Therefore, vitamin A deficiency induces lipid peroxidation in the sera and hearts of rats [64,65]. These findings and the fact that oxidized phospholipids formed by lipid peroxidation in membranes are biologically active *in vivo*, inducing a pattern of inflammatory genes in the heart [66], may indicate that the phospholipid increased mass observed in the hearts of vitamin A-deficient rats constitutes a potential source of lipid peroxides.

Vitamin A deficiency also induces a decrease of mitochondrial AA content in the hearts of vitamin A-deficient rats. This, and the decrease of its precursor, linoleic acid [57], could be associated with an activation of delta-6-desaturase [67]. It has been shown that the mRNA levels of delta-5-desaturase, an enzyme that is involved in the desaturation pathway of essential dietary fatty acids for the production of PUFA, is more abundant in livers from vitamin A-deficient rats [68]. In addition, the expression of delta-5-desaturase is enhanced by PPARalpha [69], whose mRNA expression is increased in the heart of vitamin A-deficient rats [57].

### 3.3. Vitamin A Deficiency on Aorta Phospholipid Metabolism

Changes in the level of phospholipids may compromise the integrity and function of cell membranes of the arterial wall. In fact, phospholipid content and synthesis increase in the aorta of rats fed a vitamin-A-deficient diet. As it occurs in liver and heart, the increased relative percentage of phosphatidylcholine in the aorta of vitamin-A-deficient rats is explained by an increased endogenous synthesis of phosphatidylcholine and a high expression of CTP: phosphocholine cytidylyltransferase-alpha mRNA [70]. Furthermore, it has been demonstrated an increased lipoperoxidation in the aorta of vitamin-A-deficient rats [71]. The increased mass of phospholipids would be expected to be a potential source of lipid peroxides.

A vitamin A-deficient diet, in addition to its prooxidative effect, influences cellular and molecular regulation of phospholipid metabolism and function, at least, in liver, heart and aorta. Probably, the sum of both, vitamin A deficiency and cardiovascular disease, can lead to worse prognosis in patients at risk.

## 4. Vitamin E: Its Effects on Membrane Phospholipids

Vitamin E is an essential micronutrient for higher mammals and its requirements in humans are limited only to alpha-tocopherol because the other forms of vitamin E are poorly recognized by the hepatic alpha-tocopherol transfer protein (TTP) and they are not converted to alpha-tocopherol by humans [72]. Vitamin E has a major function as a lipid antioxidant to protect polyunsaturated membrane lipids against free radical attack [73]. Furthermore, vitamin E has non-antioxidant functions [74]. The different behaviors of vitamin E isomers might be related, at least in part, to their specific effects in the plasma membrane.

Alpha-tocopherol shows a propensity to associate with lipid rafts. It can act as a membrane stabilizer by forming complexes with the products of membrane lipid hydrolysis, such as lysophospholipids and free fatty acids, and can directly modulate cell signaling, proliferation, and gene expression [75]. However, the very low levels of alpha-tocopherol reported for whole cell extracts question how this molecule can successfully protect the comparatively enormous quantities of PUFA-containing phospholipids found in membranes that are highly susceptible to oxidative attack. Recently, Atkinson *et al.* [76] have hypothesized that alpha-tocopherol partitions into domains that are enriched in polyunsaturated phospholipids, amplifying the concentration of the vitamin in the place where it is most needed. These highly disordered domains depleted in cholesterol are analogous, but organizationally antithetical, to the well-studied lipid rafts. Thus, the vitamin and PUFA-containing phospholipids would co-localize in non-raft domains, which should be translated into specific biological actions.

### 4.1. Vitamin E on Phospholipids in the Cardiovascular System

It is well known that oxidative stress plays a key role in the pathogenesis of cardiovascular disease. A decrease in nonenzymatic antioxidants such as vitamin E and vitamin C has been observed in patients with acute myocardial infarction in addition to the elevation in ROS production due to the ischemic/reperfusion event that occurs in the heart [77]. In the middle cerebral artery (MCA) occlusion model of stroke in rats the acute administration of alpha-tocopherol prior to the MCA decreases the lipoperoxidation and the volume of infarction, and increases motor performance [78].

Although studies performed with cell culture and animal models suggest that alpha-tocopherol has promising antiatherosclerotic effects through its antioxidant activity, the prevention of lipoprotein oxidation and the inhibition of platelet aggregation, the results of its supplementation in humans in randomized prospective clinical trials have been disappointing [79]. Recent evidence indicates that vitamin E may introduce a circulatory risk by inducing flow-disturbing red blood cell (RBC) adherence to blood vessel wall and pro-thrombotic phosphatidylserine exposure. The translocation of phosphatidylserine to RBC surface is well-known as a potent mediator of RBC/endothelial cell adhesion, facilitating thrombus formation. Koshkaryev *et al*. [80] found that vitamin E hydrophilic analogue-Trolox- does not incorporate into cell membranes, and does not exhibit any of these effects, implying that the vitamin E effect is due to its known ability to incorporate into cell membranes. Thus, vitamin E elevates RBC/endothelial cell adhesion despite acting as an antioxidant.

Currently, the American Heart Association maintains that there are insufficient efficacy data from completed randomized trials to justify population-wide recommendations for use of vitamin E supplements in disease prevention.

### 4.2. Vitamin E on Phospholipids of the Liver

The liver is involved in the body-wide distribution of tocopherol through the hepatic alpha-tocopherol transfer protein, which stimulates secretion of the tocopherol from cultured hepatocytes to circulating lipoproteins and facilitates the transfer of tocopherol between membranes vesicles *in vitro* [81]. The alpha-tocopherol function is evident from the observations that mutations in the human and mice *ttpA* gene lead to vitamin E deficiency, accompanied with ataxia and neurological disorders [82].

The antioxidant properties of vitamin E seem to participate in improving morphological changes induced by different agents in the liver. The use of amiodarone, an efficacious antiarrhythmic agent, is limited clinically by its cytotoxicity in the form of lysosomal phospholipidosis development and by an indirect effect through membrane destabilization. Electron microscopy studies of the liver from rats that had received amiodarone and vitamin-E reveals a reduced deposition of phospholipids in the mitochondria [83].

Although many liver diseases, such as autoimmune liver diseases, viral hepatitis, alcoholic liver disease and cirrhosis (any aetiology) have been associated with oxidative stress, meta-analysis studies have not found evidence to support or refute antioxidant supplements in patients with liver disease [79,84].

### 4.3. Vitamin E on Phospholipids of the Lung

Alveolar type II cells play a central role in the biosynthesis of surfactant lipids and in the assembly of the alveolar surfactant. Lung surfactant consists of 90% phospholipids and 10% proteins. Its main component, responsible for its surface tension lowering properties, is dipalmitoylphosphatidylcholine [85]. In addition to dipalmitoylphosphatidylcholine, surfactant contains cholesterol and polyunsaturated phospholipids.

In the lung, polyunsaturated phospholipids species are exposed to strongly oxidizing conditions. The fatty acids are exposed directly to oxidative air pollutants which are produced by activated neutrophils and macrophages [86]. Oxidation leads to degradation of the PUFA and modified proteins. This can alter the surfactant physicochemical properties. By this mechanism, loss of surfactant function could occur without a decrease in the concentration of dipalmitoylphosphatidylcholine. In lungs of cigarette smoking rats the vitamin E level is increased, rather than decreased. This suggests that vitamin E may be mobilized from other organs to the lung under oxidative stress, presumably to maintain a constant antioxidant level [87]. Antioxidant deficiency may lead to surfactant dysfunction and respiratory insufficiency [88]. Vitamin E in the lung can also exhibit non-antioxidant effects that are at least in part mediated by the modulation of the activity of protein kinase C [89].

### 4.4. Other Vitamin E Actions on Phospholipids

Most cytotoxic therapies used for cancer treatment, predominantly act by triggering apoptosis in target cells. The anticancer activity of vitamin E appears to be caused by several mechanisms, such as apoptosis and antiangiogenesis. It is known that ceramide-enriched lipid microdomains can be formed by increased amounts of ceramide in cell surface membranes which is regulated by sphingomyelinase hydrolysis of raft-associated sphingomyelin to ceramide [90]. The treatment of human MDA-MB-231 breast cancer cells with alpha-tocopherol ether-linked acetic acid (alpha-TEA), an analogue of vitamin E (RRR-alpha-tocopherol) that exhibits anticancer actions *in vitro* and *in vivo* in different cancer types, induces an increase of the acid sphingomyelinase activity and sphingomyelinase translocation from cytosol to the cell surface membrane. This leads to co-localization with the signalling mediators Fas, DR5, and FADD, followed by activation of caspases-8 and -9 and apoptosis [91]. Sphingomyelinase activation and membrane ceramide accumulation are early events that contribute to α-TEA-induced apoptosis *in vitro* and perhaps *in vivo* [91].

On the other hand, *in vitro* and *in vivo* experimental evidence indicates that tocotrienol, an unsaturated version of vitamin E, exhibits anti-angiogenic properties that may assist in tumor regression. The antiangiogenic effects of tocotrienol might be associated with changes in phosphatidylinositol-3 kinase (PI3K)/PDK/Akt signaling and apoptosis induction in endothelial cells [92].

Although the above experimental evidences show that non-antioxidant effects of vitamin E and analogs are associated to phospholipids metabolism, further experimental and clinical investigation of the molecular events induced by vitamin E on phospholipid metabolism and signaling are required to confirm a beneficial effect of prolonged administration of high-dose natural vitamin E in reducing cancer risk or on the progression of this disease.

## 5. Folates: Its Effects on Phospholipid Metabolism

Folates are members of the B-complex vitamins, which are required for the one-carbon transfer reactions necessary for DNA synthesis and biological methylation of a wide variety of essential biological substances, including phospholipids, proteins, DNA, and neurotransmitters, thus regulating their function [93]. As mammals are unable to synthesize folate *de novo*, the requirements must be satisfied from nutritional sources. Folates are hydrophilic anionic molecules that can only minimally traverse biological membranes by simple diffusion, so there exists a well-developed folate membrane transport system for the absorption of folate in the intestine [94] and for its uptake in the different tissues. Any impairment in these folate transport systems or folate metabolism might lead to a state of folate deficiency [95]. Recent evidence suggests that acute folate over supplementation results in a significant decrease in intestinal folate uptake by down-regulating the expressions of folate carrier (RFC) and proton-coupled folate transporter (PCFT) [96].

The folate pathway is closely linked to the formation of homocysteine and metabolism of methionine and S-adenosylmethionine (SAM), two compounds that play a central role in biologically important methylation reactions. SAM is the methyl donor for >100 different transmethylation reactions [97]. The most studied in the area of lipid methylation are the three successive methylation steps of phosphatidylethanolamine resulting in the formation of phosphatidylcholine, which is known to increase membrane fluidity. Methylation reactions are required for the synthesis of membrane phospholipids, myelin basic protein and neurotransmitters, three important pathways in the central nervous system. It has been reported that individuals who are severely deficient in 5,10-methylenetetrahydrofolate reductase (MTHFR) activity have excessive amounts of homocysteine in the blood, developmental delay and vascular diseases [98]. Older heterozygous and homozygous knockout mice have abnormal lipid deposition in the proximal portion of the aorta that is consistent with the proposed role of MTHFR deficiency in vascular disease [99]. Modification of sarcolemmal phosphatidylethanolamine *N*-methylation has been observed during heart hypertrophy [100].

Folate intake/status may also influence choline metabolism/status. Choline is a fundamental nutrient for the normal function of all cells. In addition to being the precursor for acetylcholine synthesis, it is a major methyl group donor [101]. It is required in the synthesis of phospholipids phosphatidylcholine, lisophosphatidylcholine, choline plasmalogen, and sphingomyelin [102,103]. In humans, it has been shown that (1) choline is used as a methyl donor when folate intake is low; (2) the *de novo* synthesis of phosphatidylcholine, is insufficient to maintain choline status when intakes of folate and choline are low, and (3) dietary choline is required by adults in an amount >250 mg/day to maintain plasma choline and phosphatidylcholine, when folate intake is low [104]. These results confirm some aspects of the metabolic interdependence of folate and choline previously demonstrated in rats.

In pre-menopausal women, plasma phosphatidylcholine, declined in response to folate restriction and increased after folate treatment [105]. Notably, these changes occurred under conditions of steady choline intake (349 mg/d) and were probably due to changes in the availability of folate derived one-carbon units required for the biosynthesis of phosphatidylcholine, through the PEMT pathway. Pemt(-/-) mice fed a choline-deficient diet develop rapid steatohepatitis leading to liver failure. It has been demonstrated that liver failure in choline-deficient Pemt(-/-) mice is due to loss of membrane integrity caused by a decreased phosphatidylcholine/phosphatidylethanolamine ratio. In addition to folate intake, MTHFR C677T genotype is a strong genetic modifier of folate and homocysteine status and may influence choline status [105,106].

Studies in rodents suggest that dietary intake of choline early in life can diminish the severity of memory deficits in aged animals. Supplemental choline during pregnancy results in life-long enhancement of hippocampal function in offspring [107] and the rate of apoptosis in fetal hippocampus is inversely related to the dietary choline intake of the rat dam [108]. The induction of apoptosis is caused by a decrease in membrane phosphatidylcholine concentration [109] because this choline ester is needed for normal progression through the cell cycle [110]. Choline insufficiency is considered to be rare in humans and is manifested only during pregnancy, lactation, or starvation/fasting, because normal diets contain sufficient choline [111,112].

On the other hand, chronic ethanol consumption coupled with folate deficiency leads to rapid liver fat accumulation and progression to alcoholic steatohepatitis. An *in vivo* examination of liver lipid metabolism confirms that both increased *de novo* lipogenesis (e.g., lipid synthesis) and altered phospholipid metabolism (e.g., lipid export) contribute to the excessive accumulation of lipids in liver affected by steatohepatitis. In fact, increased liver triglyceride content in micropigs fed a 40% ethanol folate-deficient diet without SAM supplementation was accompanied by increased flux through the stearoyl-CoA desaturase pathway as indicated by increases in the ratios of [16:1(*n*_7_)] to 16:0 and [18:1(*n*_9_)] to 18:0 and suppressed flux through the fatty acid elongation and PEMT pathways compared with controls. SAM supplementation attenuated the triglyceride accumulation associated with alcohol [113]. Importantly, MTHFR polymorphism and ethanol intake have been associated with colorectal cancers [114].

Most of the effects of folic acid deficiency are associated to alterations in phospholipid metabolism. Since the introduction of folic acid food fortification throughout the world, the prevalence of folic acid deficiency has decreased. However, people with excessive alcohol intake and malnutrition are still at high risk of this deficiency.

## 6. Zinc on Phospholipid Metabolism

Zinc is an essential dietary trace element that has attained prominence in human nutrition and health. Zinc deficiency is common in infants and young children in developing countries and leads to stunted growth, increased risk of infection, and possibly poor neurodevelopment [115]. Zinc is a constituent of hundreds of proteins with catalytic and structural roles and is involved in intermediate metabolism, hormone secretion pathways, and immune defense [116,117]. It is also an essential component of many transcription factors, suggesting that alterations in Zn status are immediately translated into changes in gene expression [118,119]. Because Zn-deficiency symptoms very much resemble those of essential fatty acid deficiency, a close link between fatty acid metabolism and Zn status has been proposed [120,121].

### 6.1. Zn Deficiency on Phospholipids of the Lung

Reports on the effects of Zn deficiency on lipid metabolism in the respiratory system are relatively few. This metal ion has a number of properties which potentially enable it to modulate the function not only of airway epithelium, but also of the cells that interact with this tissue. The major phospholipid component (at the air-liquid interface) is phosphatidylcholine (70–80%) and numerous reports indicate that changes in phosphatidylcholine metabolism are a common feature of the pathophysiology of experimental chronic lung injury or adult respiratory distress syndrome. A moderate Zn deficiency (*in vivo*) can induce a physiological stimulus that enhances phospholipid synthesis and changes especially the pattern of phospholipids in adult rat lung [122,123].

The amount of 1-acyl-sn-glycerol-3-phosphate and the diacylglycerol acyltransferase expression decreases significantly in Zn-deficient lung, while phosphatidylcholine increases. To date, little is known about the mechanism of potential coregulation of triglyceride and phospholipid metabolisms. Caviglia *et al*. [124] provided evidence that triglyceride synthetic enzymes such as mitochondrial glycerol-3-phosphate acyltransferase may indirectly regulate phospholipids synthesis by controlling the availability of mainly fatty acids and diacylglycerol for phospholipids formation.

Also, Zn deficiency is accompanied by an important oxidative and nitrosative stress associated with significant morphological changes in lung parenchyma [125,126]. In addition, Frey *et al*. [127] demonstrated that fragmented phospholipids increase in the lung of patients with adult respiratory distress syndrome [127]. Measurement of antioxidants and the concentration of PUFAs in relation to phospholipid- and protein-oxidation products of lung surfactant should be useful as early parameters of surfactant dysfunction.

### 6.2. Other Effects of Zn on Phospholipids

Considerable evidence suggests a linkage between dietary deficiency of Zn and diverse alterations in the absorption of vitamins in the intestinal lumen. The nutritional status of Zn influences the plasma and tissue levels of vitamins A and E in animals and humans [128]. Intestinal absorption of fat, retinol, and alpha tocopherol has been shown to be impaired in rats fed a low-Zn diet. Under the conditions of matched food intakes and body weights between rats fed a Zn-deficient diet and those fed an adequate Zn diet, the intestinal absorption of vitamin A was lowered significantly in Zn-deficient rats [129]. The lower vitamin absorption levels in Zn-deficient rats occurred in parallel with a significant decrease in ^14^C-oleic acid absorption. Noh *et al*. [130] showed that the luminal hydrolysis of phosphatidylcholine to lisophosphatidylcholine by phospholipase A2 may be impaired in Zn-deficient rats, resulting in impaired intestinal absorption of fat and fat-soluble vitamins. A possible defect in phospholipase A2 produced by Zn deficiency may limit the availability of lisophosphatidylcholine, which in turn slows the rate of intestinal chylomicron formation and absorptions of lipids and lipid-soluble vitamins.

It has been observed in liver of Zn-depleted rats that content of triacylglycerol and proportions of *cis*-9-oleic, *cis*-11-vaccenic, caprylic, myristic, alpha-linolenic, and eicosapentaenoic acids increases, while stearic and arachidonic acids decrease. Also, Zn deficiency reduced fatty acid oxidation. These changes produce modification in the composition of phospholipids [131]. Alterations in the expression of gene groups functionally linked to hepatic lipogenesis and lipolysis are involved in the above lipid modifications. An unbalanced gene transcription control via PPARalpha and SREBP-dependent pathways could explain most of the apparently pleiotropic effects of Zn deficiency on fat metabolism, especially in the liver [131].

Merrells *et al*. [132] investigated the effect of dietary Zn deficiency on male reproductive system. Growing rats with severe Zn deficiency had a lower testis, seminal vesicles and prostate weights, and abnormal sperm morphology than control rats. The dominant fatty acid in testes of Zn-deficient rats was docosapentaenoic acid, comprising 15 and 24% of phosphatidylcholine and lisophosphatidylethanolamine, respectively. Thus, severe Zn deficiency during sexual maturation adversely affects sperm integrity and alters phospholipid fatty acid composition in testis by interrupting essential fatty acid metabolism. It has been suggested that Zn deficiency-associated abnormal testicular function is perhaps preceded by alterations in phospholipid fatty acid composition [132].

Furthermore, Zn has been implicated in altered adipose metabolism, insulin resistance and obesity. Tallman *et al.* [133] studied the effects of dietary Zn deficiency and supplementation on adiposity, serum leptin and fatty acid composition of adipose triglycerides and phospholipids in C57BL/6J mice fed low-fat or high-fat diets for a 16 week period. Dietary fat, but not dietary Zn, altered the fatty acid composition of adipose tissue phospholipid and triglyceride despite differences in Zn status. The high fat-fed mice had reduced adipose Zn concentrations, higher percentage of AA, elevated ratio of (*n*−6)/(*n*−3), lower ratio of PUFA/SAT and reduced percentage of total (*n*−3) fatty acids in adipose phospholipids, a fatty acid profile associated with obesity-induced risks for insulin resistance and impaired glucose transport. Furthermore, serum leptin concentration was positively correlated with body weight and body fat, and negatively correlated with adipose Zn concentration. These results support an interrelationship among obesity, leptin and Zn metabolism [133].

## 7. Magnesium on Phospholipids Metabolism

### 7.1. Magnesium on Phospholipids of the Cardiovascular System

There exists a considerable amount of experimental, epidemiological and clinical evidence that suggests a linkage between Mg dietary deficiency and diverse types of cardiovascular alterations. A low Mg content in drinking water found in areas of soft water and Mg-poor soil is associated with high incidence of ischemic heart disease, coronary vasospasm, and sudden cardiac death [134,135]. At present, the average dietary intake of Mg has declined from about 450–485 mg/day in 1900 to about 185–235 mg/day for large segments of the North American population [136].

Dietary Mg deficiency results in falls in myocardial glycogen, glucose-6-phosphate, glycerol phosphate, as well as the contents of phosphatidylcholine, phosphatidylethanolamine, diphosphatidyl glycerol, phosphatidylinositol and total phospholipid phosphorus. The mitochondrial oxidation of long-chain fatty acids is also altered after Mg depletion. These observations are consistent with the tenet that prolonged low [Mg^2+^] zero can result in marked reduction in oxygen and substrate delivery to the cardiac myocytes, with concomitant changes in membrane phospholipids [137].

Furthermore, a short-term Mg dietary deficiency (21 days) in rat results in an upregulation of the two major subunits of serine palmitoyl-CoA-transferase, serine palmitoyl transferase (SPT 1) and SPT 2 (the rate-limiting enzymes responsible for the *de novo* biosynthesis of ceramides), concomitant with a highly significant release of mitochondrial cytochrome *c* in left ventricular, right ventricular, atrial, and abdominal aortic smooth muscle [138]. This nutritional deficiency can cause a biosynthesis of ceramides via two pathways in cardiovascular tissues, viz., via the activation of serine palmitoyl-CoA-transferase and sphingomyelinase, which leads to apoptotic events via intrinsic and extrinsic pathways [138]. The dietary Mg deficiency-induced membrane oxidation leads to the generation of ceramides (via the activation of sphingomyelinase) and diminished phosphatidylcholine synthesis, which act in concert to help induce programmed cell death in ventricular, atrial, and vascular smooth muscle cells in the intact animal [139]. It has been proposed that Mg deficiency should be added to well-known extracellular stimuli such as vitamin D, TNF-alpha, FasL, IL-1β, gamma-radiation, UV radiation chemotherapeutic agents, oxidative stress, and others, which cause the activation of sphingomyelinase and hence of the sphingomyelin-ceramide pathway [140].

Mg deficiency as a risk factor for cardiovascular diseases has been related to imbalance of thromboxane and prostacyclin in the vasculature. Low level of Mg in the culture medium of human umbilical vein endothelial cells induces a time- and dose-dependent increase in AA release from the cell phospholipids, stimulates ^45^Ca^2+^ influx, resulting in an activation of phospholipase A2 (PLA2), stimulates ciclooxygenase-2 (COX-2) activity and enhances 6-keto-prostaglandin F1alpha (PGF1alpha) production [190]. The increased prostacyclin production could provide protection against the cardiovascular effect of thromboxane which is increased by Mg deficiency [141].

These results extend previous findings that low levels of Mg^2+^ in primary cultured peripheral and cerebral vascular smooth muscle cells result in lowered intracellular levels of both phosphatidylcholine and sphingomyelin-the lower the external level of Mg^2+^, the lower were the concentrations of both phosphatidylcholine and sphingomyelin [139].

### 7.2. Magnesium on Phospholipids of the Blood Components

It has been reported that weanling rats pair-fed with Mg-deficient diets for eight days exhibited an increase in fluidity of erythrocyte membranes, where a reduced ratio of sphingomyelin to phosphatidylcholine and a reduced ratio of cholesterol to phospholipid, but no change in phosphatidylcholine content, was found [142]. Altura *et al.* [140] have shown that short-term dietary deficiency of Mg in an adult rat results in decreased serum levels of both phosphatidylcholine and sphingomyelin.

Sphingomyelin and phosphatidylcholine are the major phospholipids in very low-density lipoproteins, and serum lipids of Mg-deficient rats and rabbits exhibit dramatic changes in these lipoproteins and significant elevations in serum/plasma triglycerides [143] along with increases in phospholipids [144]. It is possible that significant changes in sphingomyelin/phosphatidylcholine containing lipoproteins, and the current report of lowered serum sphingomyelin and phosphatidylcholine concomitant with lipid peroxidation, could be in part due to a redistribution of SM and phosphatidylcholine in the changing lipoprotein fractions independent of sphingomyelinase activity [140].

Mg^2+^ has been reported to increase the affinity between coagulation factors IXa and VIIIa [145] and, remarkably, it does not enhance the rate of factor X activation by IXa/VIIIa unless phospholipids are included in the system [145]. Recent evidence indicates that Mg^2+^ binding to the specific sites in the *C*-terminal half of the IX/IXa γ-carboxyglutamic acid (Gla) domain prepares it for Ca^2+^ binding to the *N*-terminal half of the Gla domain of vitamin K-dependent clotting proteins [146]. Mg^2+^ enhances factor IXa binding to phospholipids at physiological concentrations of Ca^2+^. This fact could facilitate assembly of the intrinsic tenase complex. It has been concluded that Mg^2+^ promotes phospholipid binding to all vitamin K-dependent clotting proteins [147].

### 7.3. Other Functions of Magnesium on Phospholipids

Magnesium is necessary in eukaryotic cells for phosphatidate phosphatase (PAP, 3-*sn*-phosphatidate phosphohydrolase, EC 3.1.3.4) activity. This enzyme plays a central role in the synthesis of phospholipids and triacylglycerol through its product diacylglycerol, and it also generates and/or degrades lipid-signaling molecules that are related to phosphatidate. The requirement of Mg^2+^ ions as a cofactor for PAP enzymes is correlated with the catalytic motifs that govern the phosphatase reactions of these enzymes. For example, the *PAH1*-encoded PAP1 enzyme has a DxDxT catalytic motif within a haloacid dehalogenase (HAD)-like domain [148]. This motif is found in a superfamily of Mg^2+^-dependent phosphatase enzymes, and its first aspartate residue is responsible for binding the phosphate moiety in the phosphatase reaction [149].

Furthermore, Mg deficiency increases AA in renal epithelial NRK-52E cells, where the extracellular Mg^2+^ removal elevates AA release mediated mainly by Ca^2+^-independent phospholipase A_2_ (iPLA_2_) [150]. In addition, Mg influences the profile of fatty acids and their esters in rat hepatocytes. Hepatocytes incubated with MgCl_2_ in the culture medium showed a decrease of the amount of C18:2, C18:1b and C20:4 in comparison with the control sample [151]. The experimental evidence also indicates that the *n*−6/*n*−3 fatty acid composition of the diet or the physiological factors affecting oxidative stress can alter the response of rats to marginal Mg deficiency by modifying Mg metabolism, distribution and oxidative stress indicators [152]. All these observations demonstrate a complex relationship between Mg homeostasis and phospholipid metabolism.

## 8. Conclusion

The experimental evidence presented in this review indicates that fatty acids, vitamins (A, E and folate) and essential metals (zinc and magnesium) play an important role in the phospholipid metabolism of different organs of animal or human cells. Cardiovascular and mental disease, several lung pathologies and cancer, among other human disorders, exhibit a phospholipid component in their epidemiology. For this reason, further understanding of the role played by macro and micronutrients in phospholipid homeostasis should contribute to the prevention and treatment of prevalent diseases in the world. However, further discussion and research on the interactions among dietary components, phospholipid metabolism, multiple genes, and risk factors is still required.
